# Development of Modified Farquhar–von Caemmerer–Berry Model Describing Photodamage of Photosynthetic Electron Transport in C_3_ Plants under Different Temperatures

**DOI:** 10.3390/plants12183211

**Published:** 2023-09-08

**Authors:** Daria Ratnitsyna, Lyubov Yudina, Ekaterina Sukhova, Vladimir Sukhov

**Affiliations:** Department of Biophysics, N. I. Lobachevsky State University of Nizhny Novgorod, 603950 Nizhny Novgorod, Russia; dasha-lola1997@mail.ru (D.R.); lyubovsurova@mail.ru (L.Y.); n.catherine@inbox.ru (E.S.)

**Keywords:** photosynthesis, Farquhar–von Caemmerer–Berry model, photodamage, temperature dependences, thermal tolerance

## Abstract

Photodamage of photosynthetic electron transport is a key mechanism of disruption of photosynthesis in plants under action of stressors. This means that investigation of photodamage is an important task for basic and applied investigations. However, its complex mechanisms restrict using experimental methods of investigation for this process; the development of mathematical models of photodamage and model-based analysis can be used for overcoming these restrictions. In the current work, we developed the modified Farquhar–von Caemmerer–Berry model which describes photodamage of photosynthetic electron transport in C_3_ plants. This model was parameterized on the basis of experimental results (using an example of pea plants). Analysis of the model showed that combined inactivation of linear electron flow and Rubisco could induce both increasing and decreasing photodamage at different magnitudes of inactivation of these processes. Simulation of photodamage under different temperatures and light intensities showed that simulated temperature dependences could be multi-phase; particularly, paradoxical increases in the thermal tolerance of photosynthetic electron transport could be observed under high temperatures (37–42 °C). Finally, it was shown that changes in temperature optimums of linear electron flow and Rubisco could modify temperature dependences of the final activity of photosynthetic electron transport under photodamage induction; however, these changes mainly stimulated its photodamage. Thus, our work provides a new theoretical tool for investigation of photodamage of photosynthetic processes in C_3_ plants and shows that this photodamage can be intricately dependent on parameters of changes in activities of linear electron flow and Rubisco including changes induced by temperature.

## 1. Introduction

Photosynthesis supports life on Earth through the fixation of solar energy [1,2], the synthesis of organic substances and the production of oxygen [3]. All photosynthetic reactions can be divided into two groups: light reactions and dark reactions. During light reactions, a complex system of membrane-associated pigments absorb light [4]; the absorbed light energy is used to separate charges in photosynthetic reaction centers and create a transmembrane electrochemical potential across thylakoid membranes [5]. An electron is transferred from water to the high-potential electron end-acceptor NADP^+^ [1]. This transfer is accompanied by the production of oxygen, which is released into the environment [6]. At the same time, ATP synthesis is carried out due to the transmembrane potential [7]. Thus, the products of light reactions are energy equivalents (ATP and NADFH [8]) and oxygen. Energy equivalents are used for the synthesis of carbohydrates from carbon dioxide (CO_2_) and water during the Calvin cycle [1]. CO_2_ used by photosynthetic dark reactions is transported into leaves from the atmosphere through the stomata [3] and then diffuses to chloroplasts. CO_2_ stomatal conductivity and the conductivity of the mesophyll of leaves affect the concentration of CO_2_ in chloroplasts [9].

Photosynthesis can be subject to the negative influence of environmental stressors (excessive light, non-optimal temperatures, drought, salinization, etc.) [10,11]. Stressors suppress photosynthetic light and dark reactions; this effect leads to a slowdown in the rate of biomass production [12]. The suppression can be based on changes in the ultrastructure of chloroplasts, in the number of pigments and metabolites, in the stomatal opening and in other parameters [6]. Particularly, photodamage of photosynthetic machinery including photosystem II [13,14] is one of key mechanisms of photosynthetic suppression because this damage can be induced by both the high activity of photosynthetic light reactions (e.g., under action of excess light [15]) and low activity of photosynthetic dark reactions (e.g., under temperatures that are not optimal for the Calvin–Benson cycle [16] or under drought, which closes stomata and decreases the CO_2_ flux into leaves and chloroplasts [17]).

The central role of photodamage in the disruption of photosynthesis highlights the importance of analyzing this process. However, the efficiency of experimental investigations is restricted by the complexity of photodamage mechanisms, which includes the simultaneous participation of photosynthetic light reactions, photosynthetic dark reactions, and CO_2_ fluxes through stomata, plasma membranes, and chloroplast envelopes in forming photodamage. These restrictions can be overcome by using mathematical modeling [18,19,20], which can simultaneously describe all processes influencing photodamage and their interactions.

Mathematical models can describe different levels of photosynthetic processes: from the reaction centers to plant canopies and ecosystems [21]. Models of stationary energy flows in reaction centers are the basis of interpreting the results of chlorophyll fluorescence measurement which is a key method used to investigate photosynthetic light reactions [8,22]. Models of the electron transport chain (ETC) in thylakoid membranes [19,23,24,25] can additionally describe the regulation of photosynthetic light reactions by some environmental factors (e.g., light). There are also models describing the assimilation of CO_2_ with a minimal description of the ETC, e.g., the Farquhar–von Caemmerer–Berry (FvCB) model which describes photosynthesis in C_3_ plants (the basic FvCB model) [26,27,28] or the model by Laisk et al. [20] which is based on a detailed description of the Calvin–Benson cycle and the simulation of relations between photosynthesis and other physiological processes (e.g., amino acid synthesis). Further development of photosynthetic models can be based on the description of spatially heterogenous processes (e.g., in leaves [29,30,31,32] or plant canopies [33]), simulation of productivity [34], modelling interactions of plants with each other and with other living organisms [18] and others.

Considering the relation of photodamage to both light and dark photosynthetic reactions, the FvCB model [26,27,28], which minimally describes both groups of reactions, seems to be a prospective tool for the theoretical investigation of light-induced damages of photosynthetic machinery under adverse environmental conditions. This model is based on a description of limitations of photosynthetic CO_2_ assimilation using the “slowest” processes including the rate of CO_2_ carboxylation/oxygenation by Rubisco (RuBP), rate of ETC activity and rate of triose transport in the basic variant of the FvCB model [35]. There are numerous modifications to the FvCB model; particularly, it can include descriptions of cyclic and pseudocyclic electron flows, CO_2_ fluxes from air to the stroma of chloroplasts [28] including spatially heterogenous fluxes [31,32], temperature dependences of photosynthetic processes [35,36], etc. The latter descriptions can have especial importance regarding photodamage under action of non-optimal temperatures because low or high temperatures can stimulate light-induced damage of photosynthetic machinery [37]; however, the FvCB model and its modern modifications are not often focused on descriptions of photodamage including theoretical analysis of temperature dependences of this damage.

Thus, the aim of the current work was the development of a modified FvCB model describing photodamage of photosynthetic electron transport in C_3_ plants under different temperatures. Pea plants are used as the research object for parameterization of the model to minimize the variability of its parameters; however, results of the model analysis should be qualitatively similar to the results of simulation concerning parameterization on the basis of other C_3_ plants. It is expected that this model can be used as a relatively simple theoretical tool for understanding the characteristics of light-induced damages of photosynthetic machinery under action of adverse temperatures.

## 2. Modified Farquhar–von Caemmerer–Berry Model with Description of Photodamage

### 2.1. General Description of the Model and Equations

**Figure 1:**
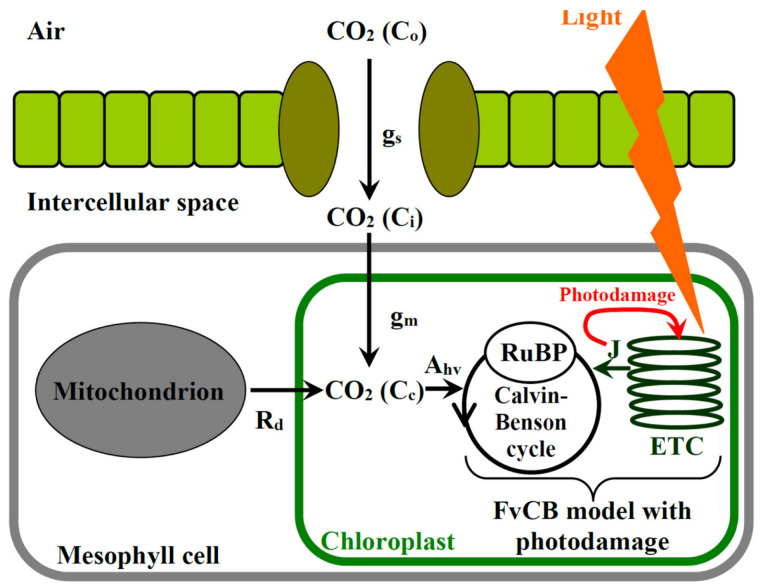
The general scheme of the modified FvCB model describing photodamage of photosynthetic electron transport (see text for details).

Figure 1 shows the general scheme of the developed mathematical model of photodamage of photosynthetic electron transport in C_3_ plants. Our model was based on the Farquhar–von Caemmerer–Berry model [26,35,38]. The steady-state rate of photosynthetic CO_2_ assimilation was described as being equal to the slowest of the two main processes. The first process is carboxylation/oxygenation, related to RuBP activity. This process was limiting under the low CO_2_ concentration in the modeled chloroplast. The second process is the regeneration of RuBP, associated with the production of NADPH from NADP^+^ through photosynthetic light reactions. This regeneration was taken as a function of the potential rate of the linear electron flow through the ETC under the specific intensity of actinic light (J, µmol∙m^−2^∙s^−1^). In accordance with a series of works [31,32,36], we did not include the limitation of photosynthetic CO_2_ assimilation by the rate of triose transport because it was difficultly distinguished from the RuBP carboxylation/oxygenation limitation [36].

Thus, Equations (1) and (2) were used for the description of photosynthetic CO_2_ assimilation (A_hv_):

(1) $$W=min\left(W_{j},W_{c}\right)$$

(2) $$A_{hv}^{}=W\cdot \frac{C_{C}-\Gamma^{*}}{C_{C{}_{}}}$$

where W is the real CO_2_ carboxylation rate (µmol∙m^−2^∙s^−1^), W_j_ is the potential CO_2_ carboxylation rate under the limitation caused by ETC activity, W_c_ is the potential CO_2_ carboxylation rate under the limitation caused by RuBP carboxylation/oxygenation, C_c_ is the concentration of CO_2_ in the chloroplast (ppm) and Γ* is the photosynthetic CO_2_ compensation point (ppm).

Equation (3) describes W_j_ which is dependent on the potential rate of linear electron flow through the ETC:

(3) Wj=J4+8·Γ*Cc

In accordance with the FvCB model, J was approximated by a function which depended on the absorbed energy of the actinic light (Equation (4)) [35,39]:

(4) $$J=\frac{I_{2}+J_{max}-\sqrt{{\left(I_{2}+J_{max}\right)}^{2}-4\cdot \theta \cdot I_{2}\cdot J_{max}}}{2\cdot \theta {}_{}}$$

where θ is an empirical curvature factor, J_max_ is a maximum electron transport rate through the ETC (µmol∙m^−2^∙s^−1^) and I_2_ is the absorbed light radiation by photosystem II (PSII) (µmol∙m^−2^∙s^−1^) [35].

I_2_ was described as a constant fraction of the actinic light [40] (Equation (5)):

(5) $$I_{2_{}}=PAR\cdot \beta^{\prime }$$

where PAR is the actinic light intensity and β’ is a coefficient calculated as the multiplication of the fraction of light absorbed by the modeled leaf and the fraction of light energy directed to the PSII.

W_c_ was calculated on basis of the Equation (6):

(6) Wc=CC·VcmaxCC+Kc·(1+OKo)

where V_cmax_ is the maximum rate of RuBP activity (µmol∙m^−2^∙s^−1^), K_c_ and K_o_ are the Michaelis–Menten constants of carboxylation and oxygenation (ppm^−1^) and O is the O_2_ concentration (ppm) [35].

The rate of carboxylation depended on the concentration of CO_2_ inside the chloroplast. CO_2_ was transported into the chloroplast from the air through the leaf tissue. Also, CO_2_ entered from the mitochondria, where it was produced by respiration (R_d_, µmol∙m^−2^∙s^−1^). The change in CO_2_ concentrations in the intercellular space was described as:

(7) $$\frac{dC_{i}}{{dt}_{}}=g_{s}\left(C_{o}-C_{i}\right)-g_{m}(C_{i}-C_{c})$$

where C_o_ and C_i_ are concentrations of CO_2_ in the air and in intercellular space, respectively; gm (mol∙m^−2^∙s^−1^) and gs (mol∙m^−2^∙s^−1^) are the mesophyll conductance and stomatal conductance to CO_2_, respectively.

After that, we calculated the changes in the concentration of CO_2_ under both conditions: (i) the limitation caused by the rate of CO_2_ carboxylation by RuBP (Equation (8)) and (ii) the limitation caused by the rate of electron flow through the ETC (Equation (9)):

(8) dCccdt=gmgs·Co+gm·Ccgs+gm−Cc−Cc−Γ*·VcmaxCc+Kc·1+OKo+Rd

(9) $$\frac{dC_{c}^{j}}{dt}=g_{m}\left(C_{i}-C_{c}\right)-\frac{\left(C_{c}-\Gamma^{*}\right)J}{4\cdot C_{c}+8\cdot \Gamma^{*}}+R_{d}$$

In our model, we calculated stationary $C_{c}^{c}$ on the basis of Equations (7) and (8) and stationary $C_{c}^{j}$ on the basis of Equations (7) and (9). Final C_c_ was calculated as $max\left(C_{c}^{c},C_{c}^{j}\right)$; this C_c_ was used for the calculation of the final C_i_ using Equation (7).

It is known that light (especially blue light) can strongly activate H^+^-ATPase in the plasma membrane [41] and activation of this transporter can increase the mesophyll conductance to CO_2_ [42]. To describe the dependence of mesophyll conductance on PAR, we used Equation (10):

(10) $$g_{m}=\frac{∆g_{max}\cdot {PAR}^{n}}{K^{n}+{PAR}^{n}{}_{}}+g_{d}$$

where ∆g_max_ is the difference between the maximum value of the mesophyll CO_2_ conductance under light and its dark level, n is the number of quanta of blue light per receptor required for increasing g_m_, K is the intensity of light inducing 50% increase of g_m_ and g_d_ is the mesophyll conductance to CO_2_ under dark conditions. Equation (10) is based on the classical Hill equation, which describes the interaction of an enzyme with a ligand; in our case, PAR was used as an analog of the concentration of substrate in the Hill equation.

Photodamage plays an important role in the negative action of excess light and other adverse factors affecting photosynthetic processes [8,43]. As a result, we included a description of photodamage of photosynthetic electron transport in our model. This damage can be associated both with the direct action of light and with the production of reactive oxygen species (ROS) [44], which destroy the enzymes of the photosynthetic apparatus [12]; however, these specific reasons were not explicitly described in our model. To describe the damage, the coefficient A_j_ was included in the model that was multiplied electron flow (J). This coefficient was the variable and was described by Equation (11):

(11) $$\frac{dA_{j}}{dt_{}}=-k_{d}^{0}\cdot (\alpha \cdot W_{j}+\left(W_{j}-W\right))\cdot A_{j}$$

where $k_{d}^{0}$ is the damage rate constant and α is the ratio of the damage rate constant at W_j_ = W to this rate constant at W_j_ > W; α, should be less than 1. It was assumed that photodamage was relatively low at W_j_ = W (W_j_ < W_c_ in accordance with Equation (1)) because all electrons transported by linear electron flow should be consumed by NADP^+^. In contrast, the damage was high at W_j_ > W_c_ because electrons could not leave the ETC and should induce overreduction of the chain in this case. To simplify model parametrization, we did not describe recovery of the damaged ETC in the model. We analyzed relatively short-term time intervals in our simulation (1 h); recovery processes require rather larger time intervals.

The model also included a block that characterized the temperature dependence of the modeled photosynthetic processes. We considered the temperature dependence of two processes: (i) the carboxylation/oxygenation by RuBP (the temperature influenced W_c_) and (ii) the linear electron flow (the temperature influenced W_j_). Both processes were described by the same empirical function (Equation (12)) with different parameters (based on [16,35,45]).

(12) $$Act=\left\{\begin{array}{c} \exp\left(-\frac{{\left(t-t_{o}\right)}^{2}}{{\sigma_{1}}^{2}}\right),t<t_{o}\\ \exp\left(-\frac{{\left(t-t_{o}\right)}^{2}}{{\sigma_{2}}^{2}}\right),t>t_{o} \end{array}\right.$$

where t is the current temperature, t_o_ is the optimal temperature for the activity of the enzyme complex (Act = 1) and σ_1_ and σ_2_ are the differences between the optimal temperature and the temperature at which the activity dropped to e^−1^. For symmetric temperature dependence, σ_1_ = σ_2_. Different coefficients Act were used for W_c_ and W_j_; W_c_ and W_j_ were multiplied by these coefficients.

Equations of the model were numerically calculated using the Euler method.

### 2.2. Experimental Procedure

Two-week-old pea seedlings (*Pisum sativum* L., cultivar “Albumen”) were used for our investigation. The plants were hydroponically cultivated under a 23 °C temperature and a 16 h (light)/8 h (dark) regime of illumination in a Binder KBW 240 (BINDER GmbH, Tuttlingen, Germany). Humidity during cultivation was not controlled. The second mature pea leaf was used for measurements.

The parametrization of the model was conducted on the basis of experimental data. Parameters of photosynthetic light reactions were measured by a Dual-PAM-100 pulse-amplitude modulation (PAM) fluorometer (Heinz Walz GmbH, Effeltrich, Germany). The main measured parameters were the quantum yields of photosystems I (Y(I)) and II (Y(II)) and the coefficient of non-photochemical quenching of the chlorophyll fluorescence (qN), which were automatically calculated using standard equations [46,47,48]. Quantum yields and light fluxes were used to experimentally calculate linear electron flows (J) (see the next section). Blue actinic light (460 nm) with different intensities was used; the 239 µmol∙m^−2^∙s^−1^ intensity was used if the light intensity is not separately specified.

Parameters of photosynthetic dark reactions were measured by a GFS-3000 infrared gas analyzer and portable gas exchange measuring system (Heinz Walz GmbH, Effeltrich, Germany). The main analyzed parameter was the photosynthetic assimilation of CO_2_ (A_hv_). A_hv_ was calculated as the difference between the CO_2_ assimilation under light and dark conditions. The content of CO_2_ in the intercellular space (C_i_), stomatal conductance to CO_2_ (g_s_) and respiration rate (R_d_) were also measured. Different external CO_2_ concentrations were used; 360 ppm was used if the CO_2_ concentration is not separately specified.

The GFS-3000 system also provided controlled temperatures and their measurements. The 23 °C temperature was used as the basic setting; to analyze temperature’s influence on photosynthetic parameters, action of other temperatures was used (10 °C, 15 °C, 30 °C, 37 °C, 41 °C, 42 °C). A humidity level of 2000 ppm was used for measurements. Dual-PAM-100 and GFS-3000 apparatus were used in combination.

### 2.3. Model Parameterization

The parametrization of our model was performed in several stages. In the first stage, we calculated the photosynthetic linear electron flow (J) using Equation (13) [49] for each light intensity used in the experiment (PAR = 108 µmol∙m^−2^∙s^−1^, 239 µmol∙m^−2^∙s^−1^, 425 µmol∙m^−2^∙s^−1^ and 758 µmol∙m^−2^∙s^−1^).

(13) $$J=I_{2}\cdot Y(II){}_{{}_{}}$$

where Y(II) is the quantum yield of PSII. Absorbed light radiation by PSII (I2) was calculated in accordance with Equation (5). β’ was calculated in accordance with Equation (14):

(14) $$\beta^{\prime }=\beta \cdot \frac{{dII}^{\prime }}{d{II}_{}}$$

where β is the average value of the coefficient in Equation (5) under a high concentration of CO_2_ (2000 ppm), dII is the fraction of the absorbed light directed to PSII under a 2000 ppm concentration of CO_2_ and dII’ is this fraction under the other CO_2_ concentrations.

Equation (15), which was derived from Equations (2) and (3) at C_c_→∞ in accordance with a previously published work [50], was used for calculation of β on the basis of A_hv_, Y(II) and PAR:

(15) $$\beta =\frac{4\cdot A_{hv}}{PAR\cdot Y(II)}$$

To estimate dII (or dII′), we used Equation (16) [34,42,50,51]:

(16) dII=1Y(II)Y(I)+1

where Y(I) and Y(II) are the quantum yields of PSI and PSII measured under a low intensity of actinic light in accordance with [50].

Thus, the experimental linear electron flow was calculated on the basis of Equation (17):

(17) $$J=\beta_{m}\cdot \frac{dII^{\prime }}{d{II}_{}}\cdot PAR\cdot Y\left(II\right)$$

**Figure 2:**
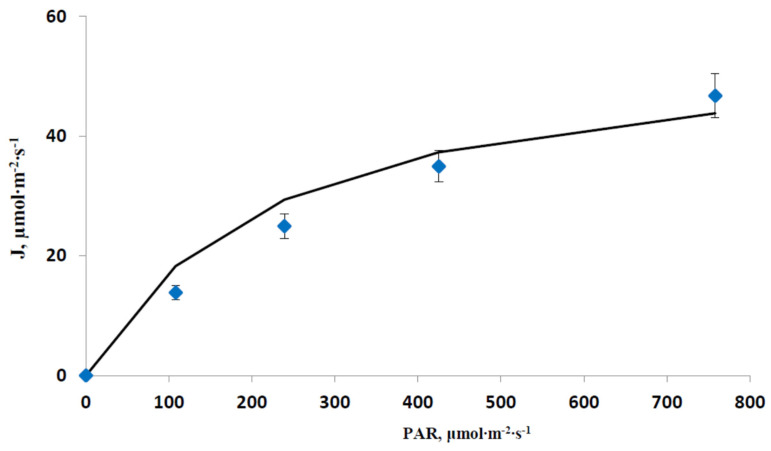
Experimental (blue markers) and model (black line) dependences of the linear electron flow (J) through the ETC on the intensity of the blue actinic light. Experimental J values were calculated on basis of Equation (17) (*n* = 7). The model dependence was approximated by Equation (4) at J_max_ = 55 µmol∙m^−2^∙s^−1^ and θ = 0.25. The determination coefficient between these dependences (R^2^) was 0.9862.

Figure 2 shows light dependences of the experimental J calculated using Equation (17) and its approximation by Equation (4) at J_max_ = 55 µmol∙m^−2^∙s^−1^ and θ = 0.25. Despite small differences between experimental and model J value, which were probably related to the simplified description of the linear electron flow in the FvCB model, the modeled J value imitated the experimental dependence well (R^2^ was 0.9862).

In the second stage of parametrization, we estimated the maximum rate of RuBP activity (V_cmax_). Based on Equations (2) and (3), $C_{c}^{j}$ (for the ETR limitation) was calculated for all actinic light intensities (Table 1). In accordance with our previous work [31], the 239 µmol∙m^−2^∙s^−1^ blue actinic light provides the limitation caused by the ETC activity in pea leaves. As a result, we used Equation (18), which was based on Equation (7), for calculation of g_m_ under the 239 µmol∙m^−2^∙s^−1^ light intensity:

(18) $$g_{m}^{}=\frac{A_{hv}-R_{d}}{C_{i}-C_{c_{}}}$$

It was calculated that g_m_ was 0.03 mol∙m^−2^∙s^−1^. We used Equation (19) for the calculation of C_c_ at C_o_ = 100 ppm and PAR = 239 µmol∙m^−2^∙s^−1^ (the RuBP limitation) on the basis of the measured C_i_ and calculated g_m_:

(19) $$C_{c}=C_{i}-\frac{A_{hv}}{g_{m_{}}}$$

It was calculated that C_c_ was 90 ppm under these experimental conditions. Using Equations (2) and (6), we calculated C_c_ and standard parameters of the FvCB model (Γ* = 38.6 ppm, Kc = 260 ppm, Ko = 179 ppm and O = 200,000 ppm [35]) and showed that V_cmax_ was 18.2 µmol∙m^−2^∙s^−1^. Using Equations (2) and (6), A_hv_, calculated V_cmax_ and noted parameters of the FvCB model, we calculated $C_{c}^{c}$ (for the RuBP limitation) under different intensities of actinic light (Table 1). The minimal rate of carboxylation corresponded to the maximum C_c_; thus, we calculated the final C_c_ as $max\left(C_{c}^{c},C_{c}^{j}\right)$ in accordance with basic equations of the FvCB model (Equations (1) and (2)).

**Figure 3:**
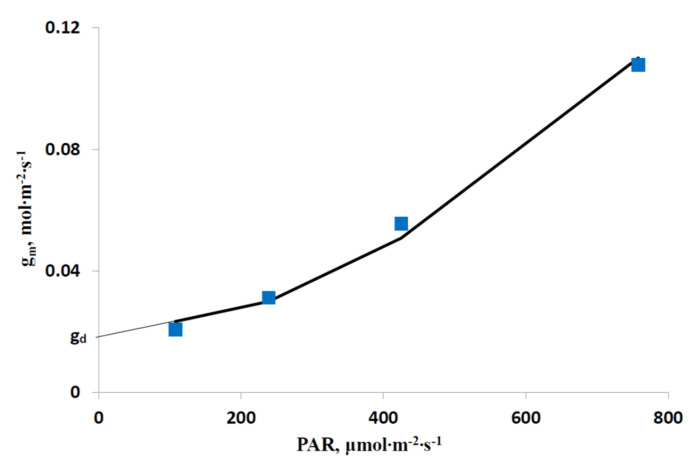
Experimental (blue markers) and model (black line) dependences of the mesophyll conductance to CO_2_ (g_m_) on the intensity of the blue actinic light. The model dependence was approximated by Equation (10) at g_d_ = 0.018 mol∙m^−2^∙s^−1^, Δg_max_ = 0.25 mol∙m^−2^∙s^−1^, *n* = 2 and K = 1000 µmol∙m^−2^∙s^−1^. The determination coefficient between these dependences (R^2^) was 0.992.

Further, we calculated experimental g_m_ under different light intensities using A_hv_, C_c_ and C_i_ in accordance with Equation (18). Figure 3 shows the experimental dependence of g_m_ on the intensity of the blue actinic light. g_m_ was increased with increases in the blue actinic light intensity which was in good accordance with the activation of H^+^-ATPase by blue light [41] and a positive influence of transporter activity on the mesophyll conductance to CO_2_ [42]. We used Equation (10) to approximate this dependence. Extrapolation of experimental g_m_ to zero showed that g_d_ was 0.018 mol∙m^−2^∙s^−1^. In accordance with [52,53], we used Δg_max_ equal to 0.25 mol∙m^−2^∙s^−1^. *n* = 2 and K = 1000 µmol∙m^−2^∙s^−1^ were assumed. The determination coefficient between experimental and model dependences (R^2^) was 0.992.

The stomatal conductance to CO_2_ (g_s_) determined by GFS-3000 measurements was relatively stable under our experimental conditions (0.17 ± 0.06 mol∙m^−2^∙s^−1^, *n* = 52). As a result, we assumed that g_s_ was constant (0.17 mol∙m^−2^∙s^−1^).

In the next stage, we parameterized the model considering the damage. For this, we used pulses of blue actinic light (70 s) with increasing intensity (PAR values were 108, 239, 425 and 758 µmol∙m^−2^∙s^−1^). Between these illumination pulses were dark intervals contributing full relaxation of the energy-dependent component of the non-photochemical quenching of the chlorophyll fluorescence; as a result, qN values before light pulses were only dependent on photodamage because participation of the state transition in non-photochemical quenching in pea plants is weak [50]. It should also be noted that in this case, only the open reaction centers of photosystem II were investigated (after the dark interval). We used Equation (20) to estimate the damage rate:

(20) $$V_{d}=\frac{{qN}_{i+1}-{qN}_{i}}{∆t_{}}$$

where qN_i+1_ and qNi are qN before initiation of light pulses i + 1 and i, respectively (after the corresponding dark intervals), and Δt is the duration of the light pulses (70 s).

Equation (21) was used for estimation of the experimental damage rate constant (kd):

(21) $$k_{d}=\frac{V_{d}}{1-qN}$$

because ${V_{d}=k}_{d}\cdot \left(1-qN\right)$. In accordance with Equation (11), ${k_{d}=k}_{d}^{0}\cdot (\alpha \cdot W_{j}+\left(W_{j}-W\right))$.

**Figure 4:**
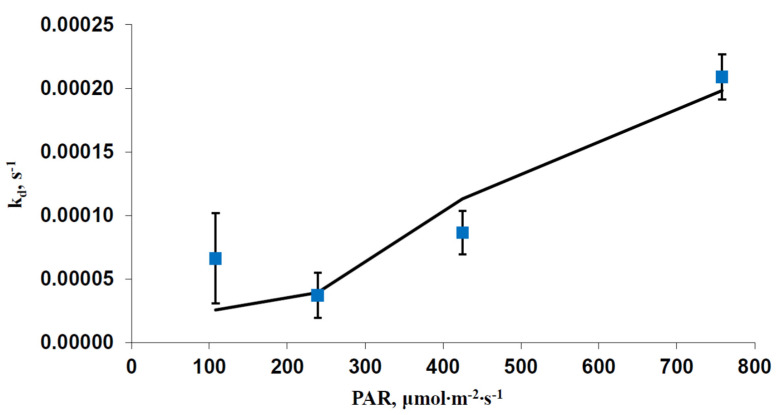
Experimental (blue markers) and model (black line) dependences of the damage rate constant (k_d_) on the intensity of the blue actinic light (*n* = 6–7). The model dependence was calculated on basis of Equation (11) at $k_{d}^{0}$ = 0.00008 s^−1^ and α = 0.09; W_j_ and W were calculated using Equations (1) and (3)–(6), standard parameters of the FvCB model [35] and results from Table 1. The determination coefficient between these dependences (R^2^) was 0.856.

Figure 4 shows the dependence of the experimental k_d_ on the blue actinic light intensity. It was shown that experimental dependence was well fitted by the model at $k_{d}^{0}$ = 0.00008 s^−1^ and α = 0.09. The determination coefficient between these dependences (R^2^) was 0.856.

**Figure 5:**
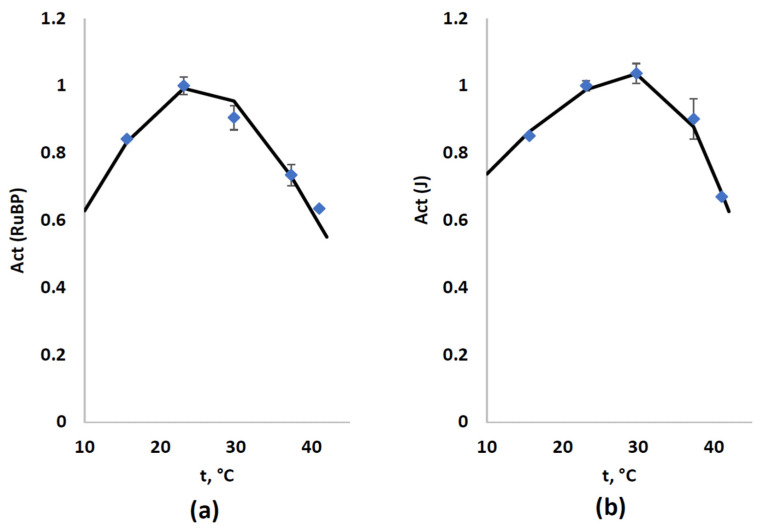
Experimental (blue markers) and model (black line) dependences of activity of the CO_2_ carboxylation by RuBP (Act(RuBP) (**a**) and linear electron flow (Act(J)) (**b**) on temperature. Experimental parameters (A_hv_ and J) were normalized on maximal values. Equation (12) was used for approximation of experimental dependences. Act(RuBP) was approximated at t_o_ = 25 °C and σ_1_ = σ_2_ = 22 °C; R^2^ = 0.942. Act(J) was approximated at t^o^ = 31 °C and σ_1_ = 36 and σ_2_ = 15.5 °C; R^2^ = 0.988.

Finally, we used experimental results to simulate temperature’s influence on both activities of RuBP (Act(RuBP) and J (Act(RuBP)) (Figure 5). We assumed in the current version of the model that non-optimal temperatures directly induced only reversible suppression of CO_2_ carboxylation and ETC activity (see Equation (12), which could not show irreversible damages); in contrast, photodamage was irreversible in our model.

It was shown that Equation (12) accurately described normalized temperature dependences of CO_2_ carboxylation by RuBP (Figure 5a) and linear electron flow (Figure 5b); parameters of these approximation and their determination coefficients are shown in the figure. It was important that the determined optimal temperatures (25 and 31 °C for Act(RuBP) and Act(J), respectively) were in good accordance with data from the literature [16].

Thus, we developed and parameterized the mathematical model of photodamage of photosynthetic electron transport which was based on the classical FvCB model. The model-based analysis of photodamage under non-optimal temperatures was the next task of our work.

The standard procedure of the model analysis included the imitation of favorable conditions (actinic light) for 20 min, imitation of stressful conditions (actinic light + transient temperature changes or transient direct decreases in RuBP activity and J) and, finally, imitation of favorable conditions (actinic light). The final damage (Aj) was analyzed after a 60 min simulation.

## 3. Results and Discussion

### 3.1. Analysis of Photodamage under Combined Decreasing Activity of Linear Electron Flow and RuBP

**Figure 6:**
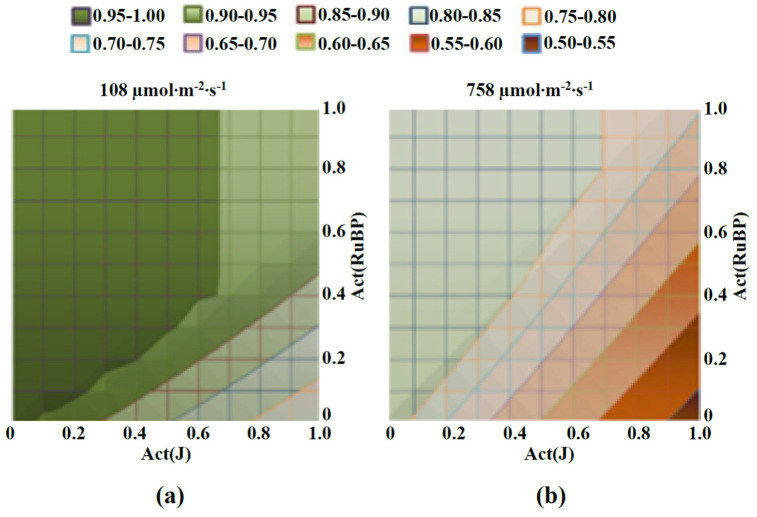
Heatmaps of the final damage of photosynthetic electron transport (the final A_j_) induced by decreasing activity of J (Act(J)) and RuBP (Act(RuBP)) under the actinic light with low (108 µmol∙m^−2^∙s^−1^) (**a**) and high (758 µmol∙m^−2^∙s^−1^) (**b**) intensities. Act(J) and Act(RuBP) in heatmaps show their values under imitation of stressful conditions. The final A_j_, which is calculated on basis of Equation (11) and can range from 0 to 1, are shown by pseudo-colors. This A_j_ shows photodamage of the electron transport because its increase corresponded to decreasing photodamage.

The analysis of the developed and parameterized model began with an assessment of the influence of a combined decrease in the activity of linear electron flow and RuBP (Figure 6). This combined decrease is probable through the influence of stressors on both processes. However, magnitudes of their decreases can differ because the sensitivity of linear electron flow and RuBP to specific stressors is different (e.g., different temperature optimums for their activity [16]). Additionally, the activity of linear electron flow can be modified by factors that do not influence RuBP (e.g., non-photochemical quenching [54] or cyclic electron flow around PSI [54]) and vice versa.

Analysis of the model showed that photodamage was simulated by both low (Figure 6a) and high (Figure 6b) intensities of actinic light under favorable conditions (Act(RuBP) = Act(J) = 1). The photodamage induction under low-intensity light was in good accordance with our previous results [25] which showed that 108 µmol∙m^−2^∙s^−1^ blue actinic light decreased qN in pea plants without action of additional stressors. This photodamage was strongly stimulated under 758 µmol∙m^−2^∙s^−1^ actinic light; it also corresponded to damage of part of the ETC by excess light [8].

Transient decreasing Act(J) and Act(RuBP) (from 20th to 40th min of simulation) strongly influenced the final A_j_ which showed photodamage (Figure 6). It was shown that the greatest damage to photosynthetic electron transport (the lowest final A_j_) was imitated under the maximum activity of the ETC (Act(J) = 1) and the minimum activity of RuBP(Act(RuBP) = 0); this effect was observed under both light intensities. In contrast, decreasing Act(J) without decreasing Act(RuBP) lowered the magnitude of photodamage imitated by the model (increased the final A_j_).

These results can be explained through suppression of the linear electron flow in this case because the high linear electron flow without sink of electrons induces overreduction of the ETC and production of ROS [25,44,55,56] that contribute to damage of both the ETC and enzymes of the Calvin–Benson cycle [57]. In contrast, the large electron sink (the active RuBP) and weak linear electron flow should minimize overreduction of the ETC and production of ROS, i.e., this combination can protect photosynthetic electron transport from photodamage.

It should be additionally noted that the simulated photodamage had thresholds in its dependences on decreasing Act(J) and Act(RuBP) under low-intensity light (Figure 6a). Particularly, under Act(J) = 1, decreasing the Act(RuBP) from 1 to 0.5 weakly influenced photodamage; in contrast, decreasing it from 0.5 to 0 gradually stimulated this damage.

Thus, results of simulation show that the transient decrease in activities of linear electron flow and RuBP strongly influences photodamage. Moreover, decreasing J can compensate for the negative influence of decreasing RuBP activity; this result is in good accordance with the positive influence of decreasing the linear electron flow on the tolerance of photosynthetic machinery (e.g., stimulation of non-photochemical quenching [54,58,59], increases in the cyclic electron flow around PSI [55] and other protective mechanisms are based on this effect). Considering the results of this stage regarding model analysis and different temperature dependences of Act(J) and Act(RuBP) (e.g., [16] or Figure 5 in the current work), it can be expected that some combinations of light conditions and values of non-optimal temperatures can decrease photodamage. Further analysis was focused on checking this hypothesis.

### 3.2. Theoretical Analysis of Temperature Dependences of Photodamage for the Different Model Parameters

**Figure 7:**
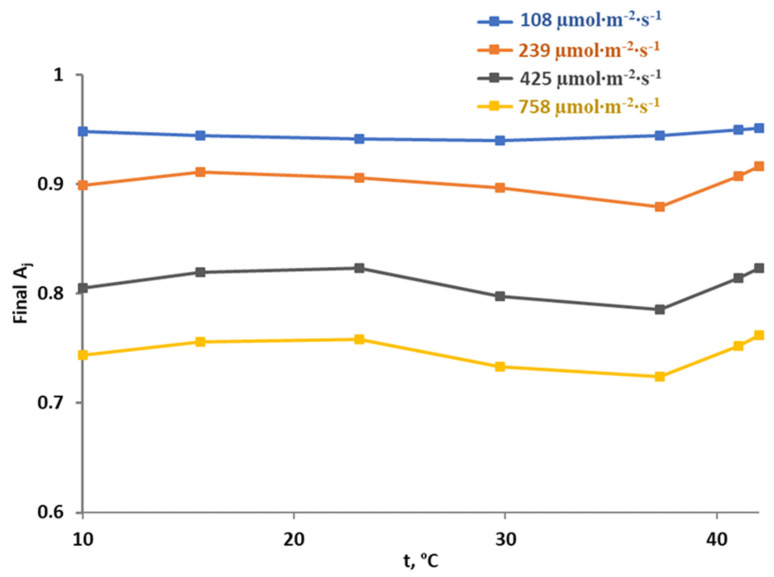
Simulated temperature dependences of the final rate of photosynthetic electron transport (A_j_) under different intensities of light for the basic model parameters. The final A_j_, which can range from 0 to 1, shows photodamage of the electron transport because its increase corresponded to decreasing photodamage.

The model-based analysis of the temperature dependence of photodamage of photosynthetic electron transport was first carried out using values of parameters which were calculated during parameterization. It was shown (Figure 7) that photodamage (decreasing the final A_j_) was induced by all simulated light intensities; magnitudes of photodamage were increased with increasing light intensity. The maximum analyzed light intensity (758 µmol∙m^−2^∙s^−1^) induced maximum photodamage (the final A_j_ was about 0.72–0.76 at the different temperatures).

Increasing temperature induced multi-phase changes which were dependent on light intensity (Figure 7). The 108 µmol∙m^−2^∙s^−1^ light induced only weak changes in the final A_j_ including decreases in this value under temperatures lower than 30 °C and increases under higher temperatures. Under 239, 425 and 758 µmol∙m^−2^∙s^−1^ light intensities, there were three phases of changes with increasing temperature: a weak increasing in final A_j_ (decreasing photodamage), a decrease in this value (increasing photodamage) and a second increase in the final A_j_ (the second decreasing photodamage).

These multi-phase dependences of photodamage can be explained by different temperature dependences of activities of linear electron flow and RuBP (Figure 5). Under low temperatures, both Act(J) and Act(RuPB) are decreased; therefore, W_j_ and W_c_ are also decreased. In accordance with Equations (1) and (11), decreasing W_j_ and W_c_ can induce opposite effects: decreasing W_j_ should decrease photodamage and increasing W_j_-W_c_ should stimulate this damage. Both processes are related to overreduction of ETC, which is the main reason behind photodamage [25,44,56], because decreasing W_j_ is related to quantity of electrons transported into the ETC, and W_j_-W_c_ is related to the balance between electrons entering the ETC and electrons leaving the ETC. Thus, weak temperature-dependent changes in the final photodamage within the 10–23 ºC temperature range are probably caused by opposite changes in the final A_j_ in this case.

The second phase of temperature dependences of photodamage (its stimulation with the temperature increasing), which is mainly observed between 23 and 37 °C, is caused by the combination of decreasing Act(RuBP) and increasing Act(J) in this temperature range, because both W_j_ and W_j_-W_c_ should be strongly increased with increasing temperature in this case. It should be noted that data from the literature [16] support different temperature optimums of activities of linear electron flow and RuBP and show that the revealed effect can be observed in other plant species.

The last phase, which is observed under increased temperatures (37–42 °C), seems to be paradoxical because increasing temperature contributes to decreasing photodamage. This result can be explained by the strong decreases in both Act(RuBP) and Act(J) with increasing temperature; it decreases both W_j_ and W_j_-W_c_ and, therefore, lowers overreduction of the ETC to protect photosynthetic machinery [56,58,59]. It should be noted that our model predicts further increases in the final Aj with strong increasing temperature (more than 42 °C; data not shown), i.e., further decreasing photodamage. However, this effect cannot correspond to real photosynthetic changes because it is known that heating to high temperatures (more than 42 °C) induces irreversible damage of photosynthetic machinery in pea plants [37]. We do not describe this direct thermal damage in the current version of our model; thus, results of analysis of this model version at high temperatures (more than 42 °C) cannot be used for prediction of photosynthetic damage in plants.

After that, we investigated the similar temperature dependences under decreasing the stomatal conductance to CO_2_ (g_s_), which differed by 10% compared to the basic g_s_. It is known [60,61] that stomatal closing is a typical plant response to action of environmental stressors including drought. This closing can disrupt CO_2_ flux from air to the chloroplast stroma and suppress photosynthetic dark reactions [31,32] stimulating damage of PSII that can protect PSI [56,62].

**Figure 8:**
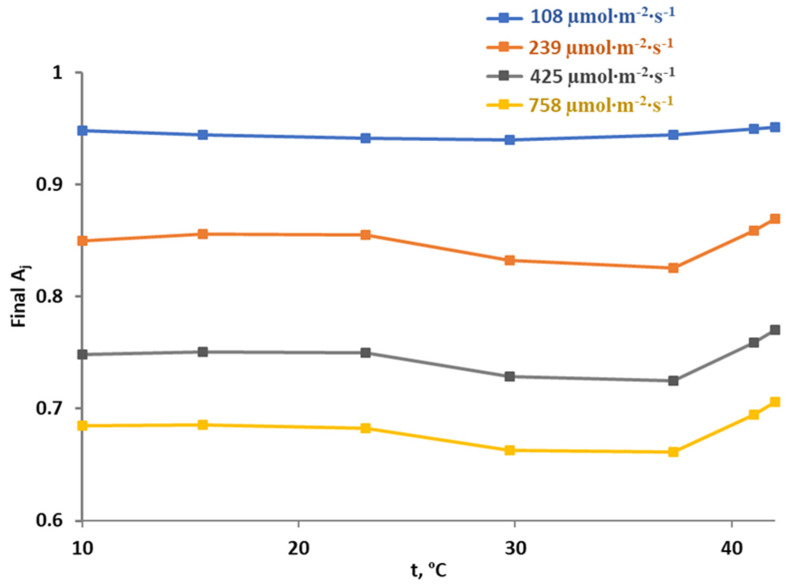
Simulated temperature dependences of the final rate of photosynthetic electron transport (A_j_) under different intensities of light at the decreased g_s_ (10% from the basic value). The final A_j_, which can range from 0 to 1, shows photodamage of the electron transport because its increase corresponded to decreasing photodamage.

It was shown (Figure 8) that decreasing g_s_ stimulated photodamage of photosynthetic electron transport and additionally lowered the magnitude of weak changes in the final A_j_ within the 10–23 °C temperature interval. In contrast, the second and third phases of changes (the increase and following decrease in photodamage) were not qualitatively modified with decreased g_s_. Thus, our model-based results show that changes in stomatal opening have a weak influence on the temperature dependence of photodamage of the photosynthetic electron flow. In contrast, the absolute values of photodamage increase with decreasing g_S_ especially, under high light intensity. This effect can be explained by a reduction in the CO_2_ flux into chloroplasts, the suppression of CO_2_ carboxylation by RuBP, and the overreduction of ETC under low g_s_. 

Changes in the thermal tolerance of photosynthetic machinery and enzymes are considered to a way of supporting productivity of agricultural plants [63,64,65], e.g., through specific regimes of fertilizer application, plant selection or methods of genetic engineering. Particularly, these approaches increase the thermal tolerance of photosynthetic machinery (see, e.g., work [64] which focused on estimation of the thermal tolerance of plant productivity in accordance with tolerance of photosystem II to heating).

**Figure 9:**
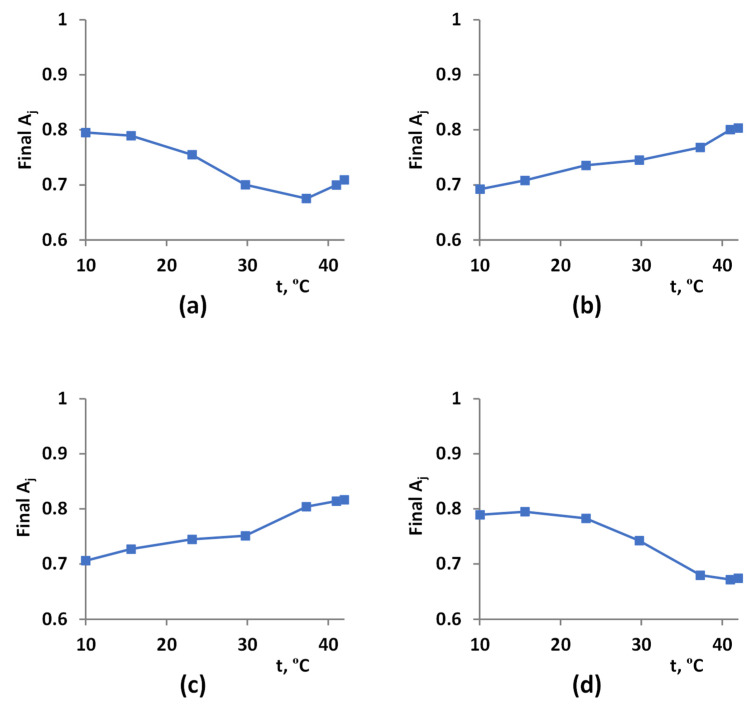
Simulated temperature dependences of the final rate of photosynthetic electron transport (A_j_) under modifications of temperature optimums of RuBP or J under the 425 µmol∙m^−2^∙s^−1^ light. (**a**) $t_{o}^{J}=31\;{}^{\circ}C$ and $t_{o}^{C}=20\;{}^{\circ}C$. (**b**) $t_{o}^{J}=31\;{}^{\circ}C$ and $t_{o}^{C}=30\;{}^{\circ}C$. (**c**) $t_{o}^{J}=26\;{}^{\circ}C$ and $t_{o}^{C}=25\;{}^{\circ}C$. (**d**) $t_{o}^{J}=36\;{}^{\circ}C$ and $t_{o}^{C}=25\;{}^{\circ}C$. The final A_j_, which can range from 0 to 1, shows photodamage of the electron transport because its increase corresponded to decreasing photodamage.

However, results of our model-based analysis (see, e.g., Figure 6) showed that the active linear electron flow in combination with suppressed RuBP activity can be more dangerous for photosynthetic electron transport than suppression of both J and RuBP. These results were in good accordance with data from the literature showing positive influence of suppression of linear electron flow on tolerance to stressors (see, e.g., works [56,62]). Thus, we analyzed the influence of changes in temperature optimums for linear electron flow and RuBP on photodamage of photosynthetic electron transport in plants in the final stage of the current investigation (Figure 9).

It was shown (Figure 9a) that decreasing the RuBP temperature optimum from 25 °C to 20 °C strongly stimulated photodamage of photosynthetic electron transport with increasing temperature. The magnitude of increase in the final A_j_ at 37–42 °C was small. Increasing the RuBP temperature optimum from 25 °C to 30 °C induced the opposite changes: the final A_j_ was increased with increasing temperature within the investigated range. However, this effect could not be interpretated as unequivocally positive for photosynthetic tolerance because the values of final A_j_ in this case were mainly lower than ones at the basic temperature optimums (see Figure 7).

Changes in the temperature optimum of the linear electron flow induced the opposite effects: decreasing this optimum from 31 °C to 26 °C was accompanied by increases in the final A_j_ (lowering photodamage) with increasing temperature and increasing the optimum from 31 °C to 36 °C was accompanied by decreases in the final A_j_ (stimulation of photodamage) with increasing temperature.

Thus, results of the model-based analysis of influence of temperature optimums on photodamage of photosynthetic electron transport show that changes in these optimums contribute to photodamage and decrease plant tolerance to the light-induced damage of photosynthetic machinery. This means that the development of new methods of crop protection based solely on changes in temperature optimums can have limited efficiency.

## 4. Conclusions

Photodamage of photosynthetic electron transport is the key mechanism of disruption of photosynthesis in agricultural plants under action of stressors which decrease their crop yield. This means that studying photodamage is an important task for basic and applied investigations. However, complex mechanisms of photodamage restrict the use of experimental methods for investigations of this process; the development of mathematical models of photodamage and model-based analysis can be used for overcoming these restrictions.

In the current work, we developed a modified FvCB model which described photodamage of photosynthetic electron transport in higher plants. This model was parameterized on the basis of experimental results (using an example of pea plants). Analysis of the model showed that combined inactivation of linear electron flow and RuBP could result in both increases and decreases in of the level of photodamage, depending on the extent of inactivation of linear electron flow and RuBP. Simulation of photodamage under different temperatures and light intensities showed that temperature dependences could be multi-phase; particularly, paradoxical increases in the thermal tolerance of photosynthetic electron transport could be observed under high temperatures (37–42 °C). Finally, it was shown that changes in the temperature optimums of linear electron flow and Rubisco could modify temperature dependences of the final photosynthetic electron transport under photodamage induction; however, these changes mainly stimulated this photodamage.

## Figures and Tables

**Table 1:** The concentrations of CO_2_ in chloroplasts (Cc), which were calculated for the J ($C_{c}^{j}$) and RuBP ($C_{c}^{c}$) limitations, and the final C_c_, which was calculated as $max\left(C_{c}^{c},C_{c}^{j}\right)$, under different intensities of the blue actinic light.

PAR, µmol∙m^−2^∙s^−1^	$C_{c}^{j}$, ppm (the J Limitation)	$C_{c}^{c}$, ppm (the RuBP Limitation)	Final (Maximal) C_c_, ppm
108	280	125	280
239	223	190	223
425	168	230	230
758	135	270	270

## Data Availability

The data presented in this study are available upon request from the corresponding author.
